# A dataset on analysis of school-based management research from 1984 TO 2023

**DOI:** 10.1016/j.dib.2024.110823

**Published:** 2024-08-20

**Authors:** Vu Thi Mai Huong, Hoang Thi Kim Hue, Dao Thi Minh Chau, Nguyen Thi Minh Nguyet, Trịnh Thi Quy, Nguyen Quoc Tri

**Affiliations:** Hanoi Nationnal University of Education, Hanoi, Vietnam

**Keywords:** Bibliometric analysis, School-based management (SBM), Scopus, VOS viewer

## Abstract

The dataset aims to map scientific publications in the field of school-based management visually at the global level indexed by Scopus using Bibliometric analysis. It focuses on articles on School-Based Management at the international level. Research data were obtained from the Scopus database from 1984 to 2023 through the publish or perish software. Then the paper will be analyzed using the VOS viewer application for viewing and creating the desired Bibliometric maps. There is a sharp increase in the number of studies on this topic, especially from 2018 to 2022 and mainly from research in the US, Hong Kong, Australia, and developed countries. Moreover, the issues of most interest are SBM in terms of student achievement, schools, Hongkong, leadership, autonomy, school autonomy, governance, school effectiveness, school improvement, school reform, decentralization, education reform, education policy, decision-making, adolescent, article, human, humans, school health services, primary education. Policymakers and scholars can find great authors, research centers, influential studies and frequently published journals on this topic to read and research. Further studies based on the combination of bibliometric analysis with other methods may help paint a more profound picture of research findings on this topic.

Specifications Table

**Table:** 

Subject	Social Sciences / Education.
Specific subject area	An analysis of school-based management research from 1984 to 2023 under bibliometric method
Data format	.csv file Raw, Analyzed, Filtered
Type of data	Table, Chart, Graph, Figure
Data collection	The dataset is based on 319 documents consisting of 78% articles, 9% reviews, 7% book chapters, 3% conference papers and 2% books, 1% editorial from 1984 to 2023 on Scopus database.
Data source location	According to the Preferred Reporting Items for Systematic Reviews and Meta-Analyses (PRISMA) standards, this study employed 348 journal articles, book chapters, conference papers, and books to search for data. The Scopus database was searched for relevant articles using the keywords “school-based management” and “> 1984 AND PUBYEAR 2023” during the identification phase. These words were filtered out of all articles' titles, abstracts, and keywords. This will make sure that the SBM's most essential materials can be found. The precise keyword to look for is “TITLE-ABS- school-based management*” ANDPUBYEAR “ > 1984 AND PUBYEAR 2023*”.
Data accessibility	Raw data were deposited at repository Mendeley data; 22 January 2024 Direct URL to data: https://data.mendeley.com/datasets/fbf4m8v2nw/1 https://data.mendeley.com/datasets/mgm78ddy5y/1

## 1 Value of The Data

• This dataset provides a comprehensive view of school-based management from 1983 to 2023. It serves as a valuable source of information that sheds light on issues related to school-based management in During the period of globalization, education in countries has strong innovation and reform towards improving quality from the school side.
• One of the main strengths of this dataset is its efficient data collection and data processing methods. It is drawn from the Scopus database. The Bibliometric method and VOS view application to directly download data contribute to its reliability, making it an indispensable tool for researchers.
• The outstanding feature of this data set is the meticulous reference of each section by country, by author, by publisher and featured authors, while also indicating the main research directions carried out creates space for further research on this issue.
• This data set provides functionality in the application. Researchers can do a lot of research from research gaps. NGOs and governments will have an important first role in reforming schools towards effective instruction.

## 2 Background

School‐based management (SBM) decentralizes decision-making and devolves responsibility to school sites within an explicit framework of both autonomy and responsibility [1]. The transition to school-based management is frequently seen as an important strategy in the present educational reform efforts to improve the effectiveness and standard of instruction in schools. Around the world, school-based management strategies have been put into place to raise educational standards in institutions of higher learning [2]. It is suggested that power decentralization, knowledge and skill development, information, and incentives be used to successfully implement programs and teach the SBM reform [3]. A school governance system called School-based management, which is founded on the ideas of school autonomy and community involvement, has been adopted in many locations throughout the world, including post-conflict countries. Such a trend appears to conform to the pattern outlined by institutional isomorphism theories. [4]. Increasing engagement and dedication as a result of empowering decision-makers at the school level has enhanced the teaching-learning environment [5].

## 3 Data Description

The dataset is classified into the proplems concluding: Topographical Landscape of the SBM and Knowledge Base; Analysis of Influential Authors and Documents; Intellectual Structure of the SBM and AS Knowledge Base.

**Fig. 1:**
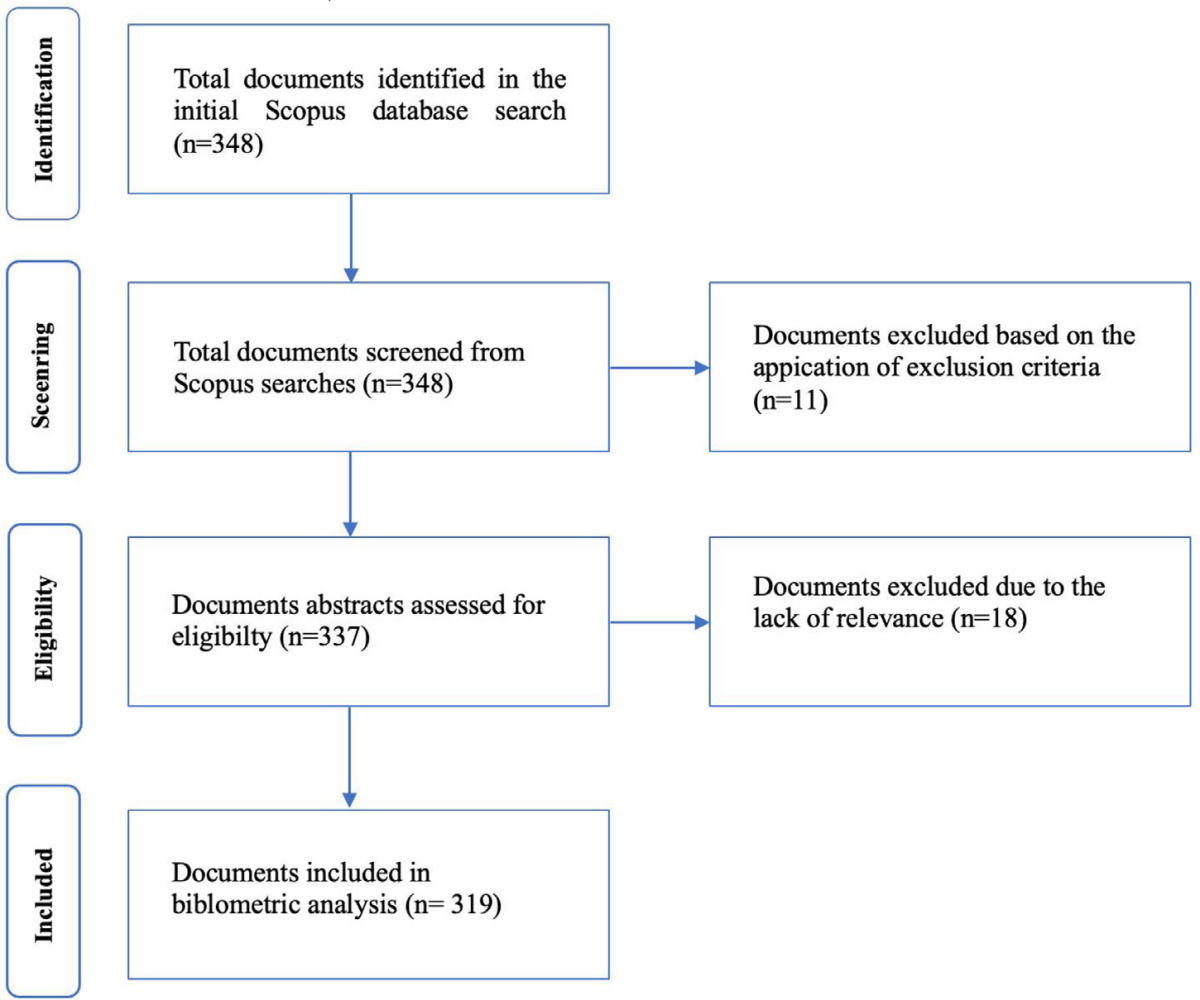
PRISMA process of determining publication dataset related to SBM (Source: Authors’ own elaboration).

**Fig. 2:**
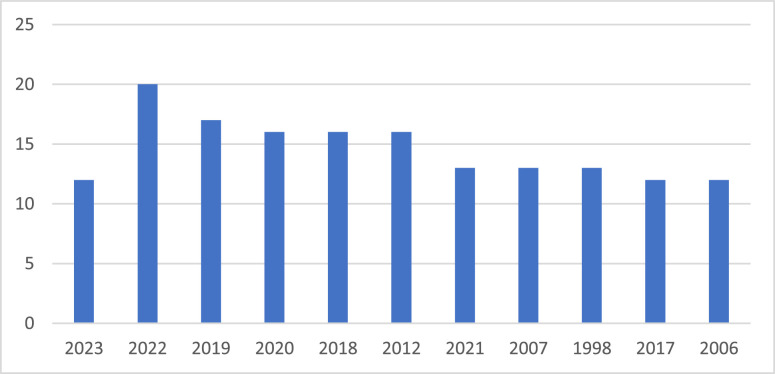
Volume growth of publications on SBM in the world from 1984 to 2023 (Source: Authors’ own elaboration).

**Fig. 3:**
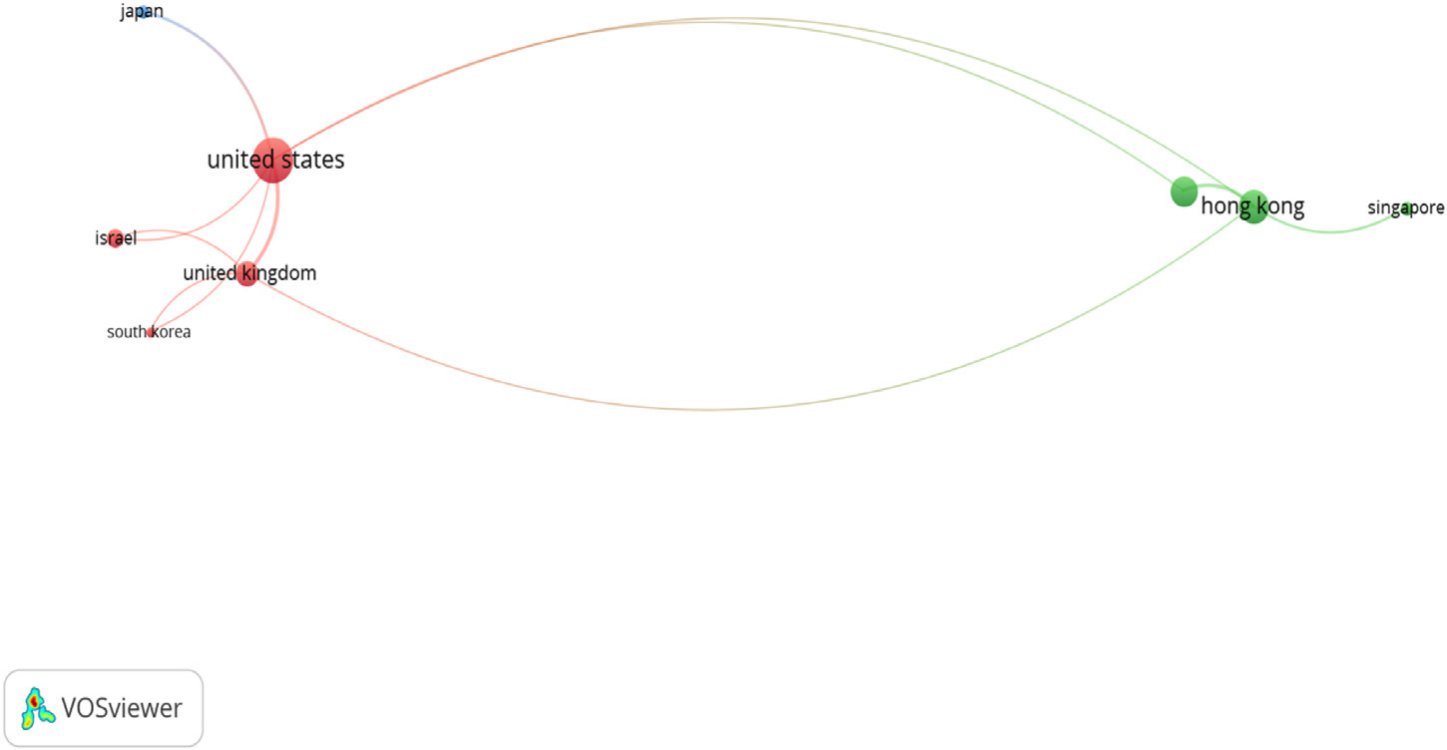
Visual representation of inter-nation co-authorship research collaboration in SBM publications (Source: Authors’ own elaboration, using VOSviewer software, the minimum number of countries for document: 5; the maximum number of countries for document: 25. Of the 55 countries, 15 meet the thresholds).

The first research problem showing in Figs. 2, 3 Tables 1, 2 focused on documenting the volume, growth trajectory, geographical distribution, and types of papers comprising the SBM knowledge base. Based on a bibliographic and content analysis of the 319 articles, we classified them by publication year, co-authors’ foreign country, institution, journal, keyword, and subfield (Fig. 1).

**Table 1:** Countries with the greatest number of SBM publications in the world from 1984 to 2023 (Source: Authors’ own elaboration).

No.	Country	Number of Publications
1	United States	84
2	Hong Kong	43
3	Australia	37
4	United Kingdom	24
5	Indonesia	23
6	Israel	15
7	Malaysia	10
8	South Africa	9
9	Canada	7
10	Japan	7

**Table 2:** Visual representation of journal cross-citation network in the research area of SBM from 1984-2023 (Source: Authors’ own elaboration, using Scopus database).

No	Source Title	Number of Publications
1	International Journal Of Educational Management	20
2	Journal Of Educational Administration	12
3	Nassp Bulletin	12
4	School Effectiveness And School Improvement	11
5	Educational Management Administration And Leadership	10
6	International Journal Of Educational Development	10
7	International Journal Of Educational Reform	7
8	Journal Of Development Effectiveness	7
9	School Leadership And Management	7
10	Australian Journal Of Education	6

The first finding is the trend in the number of papers published in the year with the largest number of publications. As shown in Fig. 2, there was a fluctuating increase in the number of papers during this period.

The year with the most publications on SBM is 2022 with 20 publications, followed by 2019 (17); 2012 (16). The year 2017 and 2006 are the lowest among the years with a high publication rate, also having 12 publications. In the past 5 years, the number of SBM studies has increased rapidly and steadily, reaching nearly 20 publications per year. Thus, more and more SBMs are interested in research.

Table 1 presents the top 10 countries with the greatest number of SBM publications in the world from 1984 to 2023. From the graph Table 1 above, it can be seen that the most research on school-based management is in the United States with 84 papers followed by Hong Kong with 43 papers. In addition, Australia, and the United Kingdom each contributed 37 and 24 papers. In Southeast Asia, Indonesia, and Indonesia each contributed 23 and 10 papers. Asia emerged with many SBM studies, including Southeast Asian countries. During this period, SBM was transferred from English-speaking countries, Europe, and North America to countries in Asia and developing countries as a way to carry out educational reforms and improve the effectiveness of the education system and the quality of education in the country.

Fig. 3 represents the recency of different countries on SBM research. The node with green and blue color implies that the authors from a respective country were involved in SBM research a long time ago (prior to 1984). The most notable node with the color green is the United States. As shown in Fig. 3, each node represents one country. The size of each node represents the quantity of SBM-related documents published by authors from the respective country. The line connecting the two nodes represents the co-authoring activities of authors from the two countries. The wider the line is, the more documents were co-authored by the respective countries. Specifically, the US had the greatest co-authorship link strength with a total link strength of 12 followed by the United Kingdom with 8, Hong Kong (8), and Australia (5). Another noteworthy point is that research collaboration through co-authorship between the US and United Kingdom, Hong Kong, Australia, Japan, Israel, and South Korea was found to be the strongest with 80 co-authored publications. Hong Kong and the USA are two countries focusing on the representation of inter-nation co-authorship research collaboration in SBM publications.

Table 2 shows a map of citation levels among journals that publish SBM. There were 10 journals with a minimum of six publications on SBM, among which the International Journal of Educational Management was the most-cited journal by others regarding this topic, after that was Journal Of Educational Administration and Nassp Bullettin.

Table 3 shows the authors' contribution to the publication of SBM since 1984. Cheng, Y.C. has published the largest number of documents with a total of 14. Edwards, D.B. has contributed the second-largest number of publications (12 documents). The rest produced 5 and 4 publications each.

**Table 3:** The 10 most published authors on SBM in the period 1984-2023.

No.	Author	Number of Publications
1	Cheng, Y.C.	14
2	Edwards, D.B.	12
3	Dimmock, C.	7
4	Cheung, W.M.	5
5	Herman, J.J.	5
6	Nir, A.E.	5
7	Wohlstetter, P.	5
8	Bandur, A.	4
9	Caldwell, B.J.	4
10	Vegas, E.Show	4

**Fig. 4:**
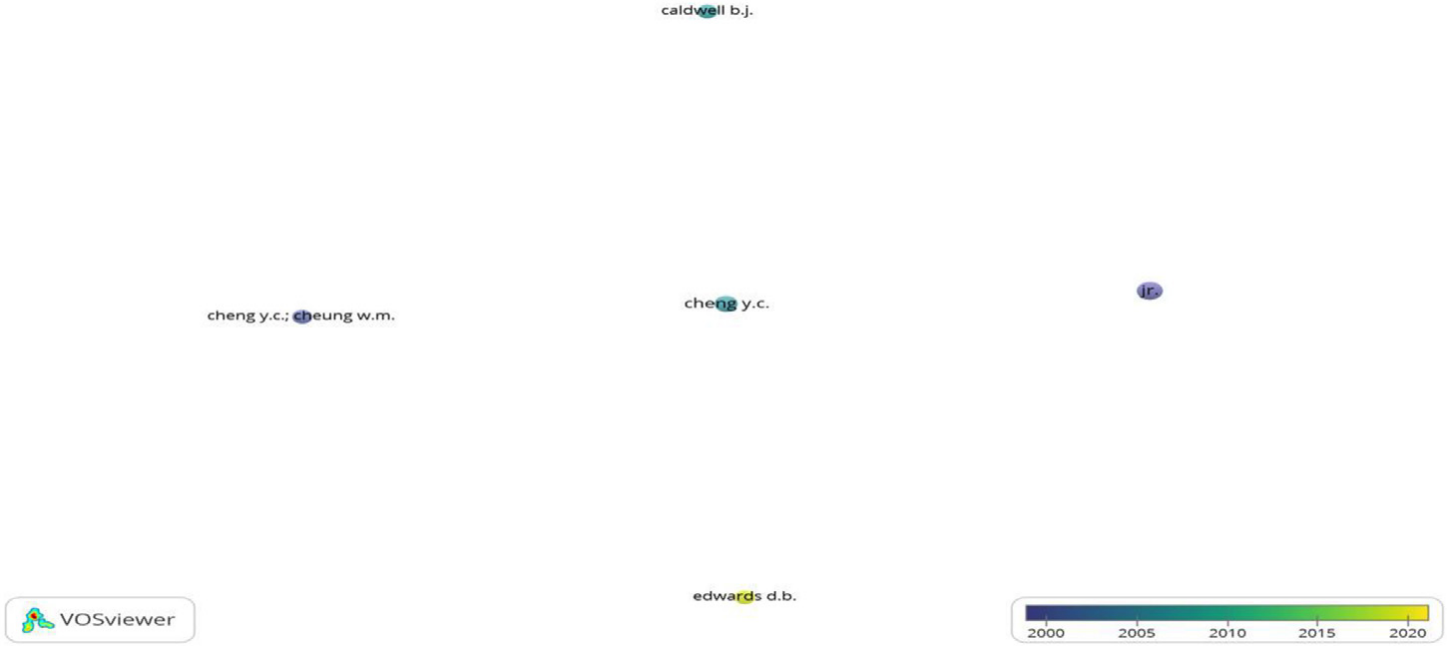
The 5 most cited authors on SBM in the period 1984-2023 (Source: Authors’ own elaboration, using VOSviewer software, the maximum number of authors per document: 25; the maximum number of an author: 4. Of the 5293 authors, 5 meet the thresholds).

In Fig. 4, among 293 authors researching SBM, 5 authors met the selection criteria. Among them, Cadwell B.J. had 51 citations from 4 documents satisfying the selection criteria of VOS Viewer Software, followed by Cheng Y.C. with 74 citations and 5 documents.

Table 4 presents the list of most cited publications on SBM. Among the most cited, the article of Marks H.M. and Louis K.S. stands out, cited 156 times. This paper is a work with respect to the association of teacher empowerment and instructional practice and student academic performance. Four publications are well cited, with more than 100 citations. Research of Ogawa R.T. and Malen B., cited 131 times, noted the authors’ experiences in reviews of multivocal literature on school-based management. The third most cited article (Dimmock C. and Walker A.) cited 130 times, proposed a framework of comparative and international educational leadership and management, comprising four elements of schooling and school-based management.

**Table 4:** Top-10 most cited publications on SBM based on Scopus database from 1984-2023.

No.	Authors	Titles	Cited by
1	Marks H.M.; Louis K.S.	Does teacher empowerment affect the classroom? The implications of teacher empowerment for instructional practice and student academic performance	156
2	Ogawa R.T.; Malen B.	Towards Rigor in Reviews of Multivocal Literatures: Applying the Exploratory Case Study Method	131
3	Dimmock C.; Walker A.	Developing comparative and international educational leadership and management: A cross-cultural model	130
4	Duflo E.; Dupas P.; Kremer M.	School governance, teacher incentives, and pupil-teacher ratios: Experimental evidence from Kenyan primary schools	124
5	Leithwood K.; Menzies T.	Forms and effects of school-based management: A review	113
6	Wohlstetter P.; Odden A.	Rethinking School-Based Management Policy and Research	77
7	Fullan M.; Watson N.	School-based management: Reconceptualizing to improve learning outcomes	77
8	Nir A.E.	School-based management and its effect on teacher commitment	67
9	Leithwood K; Menzies T.	A Review of Research Concerning the Implementation of Site-Based Management	67
10	Lingard B.; Hayes D.; Mills M.	Developments in school-based management: The specific case of Queensland, Australia	63

**Fig. 5:**
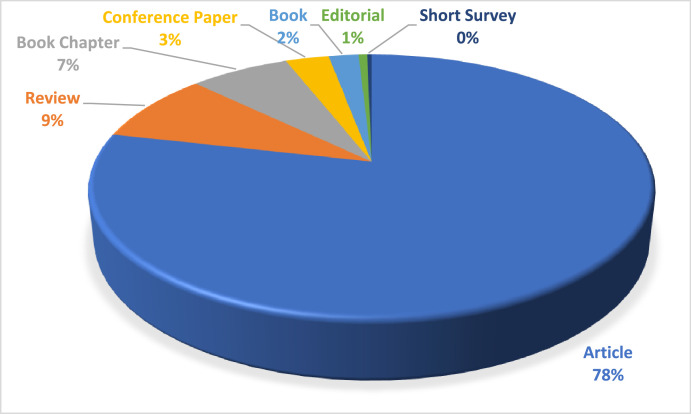
Most prominent sources by number of publications & citation count.

Fig. 5 presents the percentage of publications distributed in six different document types. Most documents were published as articles which dominates by 78 % of overall profiles. The second prominent source was Review, which dominates only by 9 %. Books, editorials, and brief surveys, which account for 2%, 1%, and 0% respectively, are the materials that come in last.

**Fig. 6:**
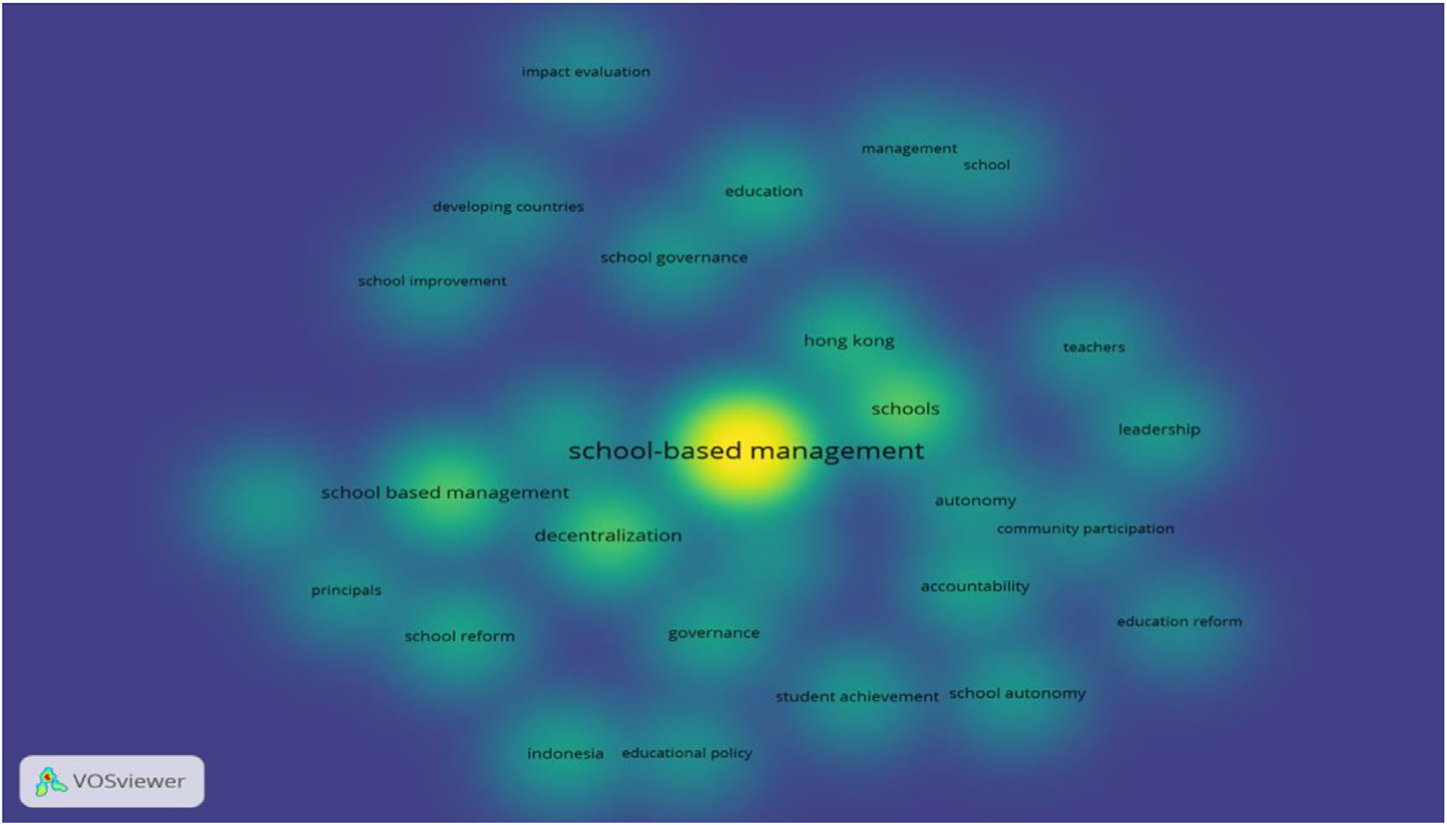
Temporal keyword co-occurrence linking with Author keywords map of the SBM literature (threshold 5 co-occurrences, display 28 documents) (Source: Authors’ own elaboration, using VOS viewer software).

Fig 6. Shows the temporal keyword co-occurrence showed co-occurrence linking with All keywords. There are 1002 keywords having 48 meet the threshold. All of the keyword distributions are ‘pushed forward’ in time. Thus, the dates on the temporal map are keyed to the most recent decade. We grouped the recently occurring keywords (yellow/light shade) into topical themes and then ranked these by the total occurrences of the composite keywords. This synthesis yielded the following themes in rank order.

**Fig. 7:**
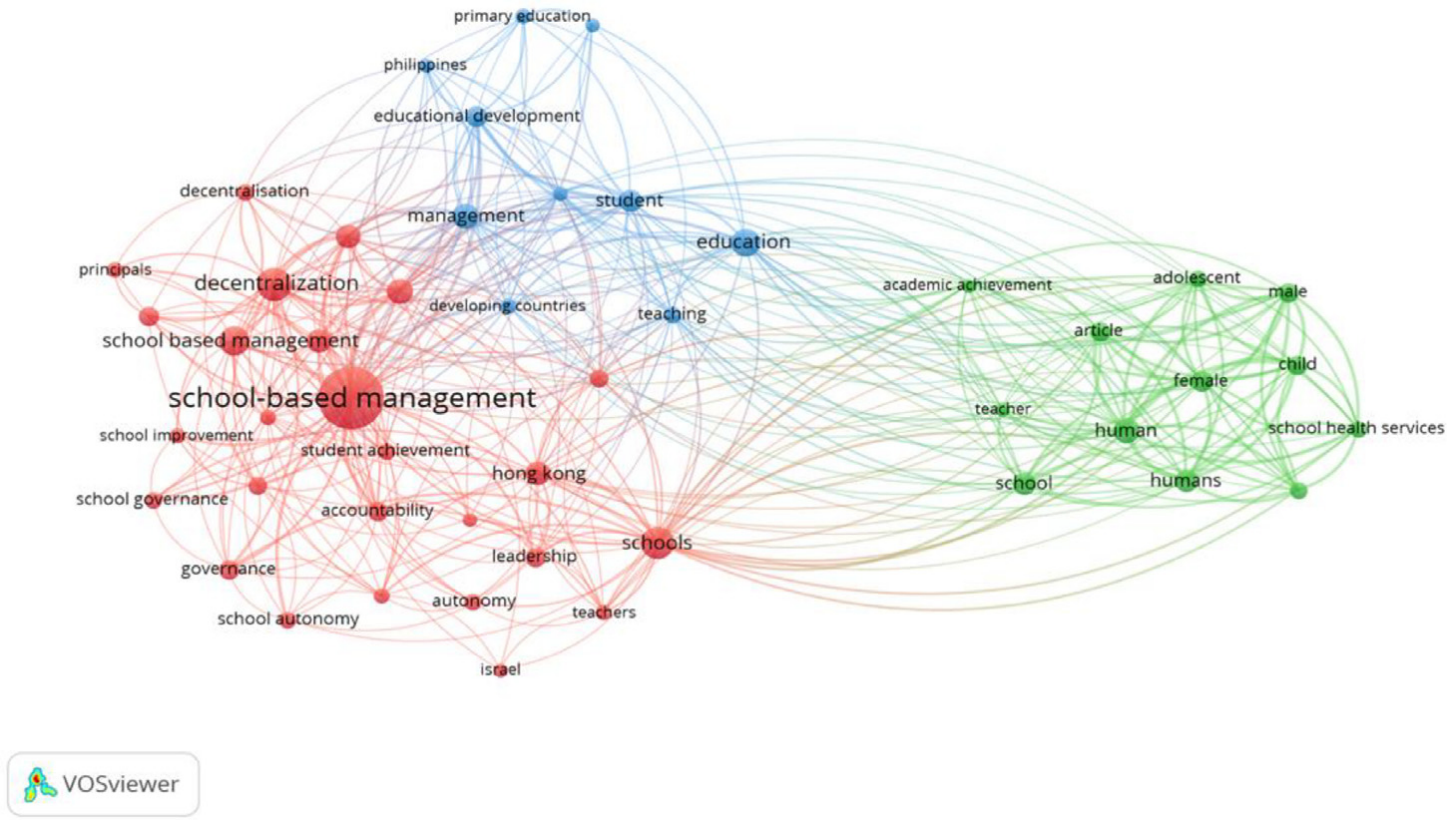
Temporal keyword co-occurrence linking with All keywords map of the SBM literature (threshold 5 co-occurrences, display 48 documents) (Source: Authors’ own elaboration, using VOSviewer software).

*School-based managenent* (student achievement, schools, Hongkong, leadership, autonomy, Israel, school autonomy, governance, school effectiveness, school improvement, school reform, decentralization, education reform, education policy, decision making, Indonesia);

*School* (teacher, academic achievement, adolescent, article, human, humans, school health services, child, male, female);

*Education development* (primary education, Philippines, management, student, education, teaching);

Fig. 7 shows the temporal keyword co-occurrence linking with All keywords map of the SBM literature (threshold 5 co-occurrences, display 48 documents) (Source: Authors’ own elaboration, using VOSviewer software). These themes highlight trends in recent research on SBM. The patterns revealed in this map offer further support for the conclusion that SBM literature is moving towards the adoption of developing countries.

This further suggests that SBM is relevant to multiple operative areas of the schools. Those are the effects of SBM on the students’ achievements (e.g., Anton De Grauwe in 2005). A school's mission, overall vision, annual programs, budget, textbooks, school building, curriculum based on school-specific teachings, and even student disciplinary policies can be established with the help of the school community by decentralizing power and authority to the school level. In exchange, empowering and authorizing the school level has brought about a number of adjustments inside the institution, including modifications to the school's culture and increased community involvement. The learning environment and student performance have improved as a result of these elements (Bandur, A., in 2012). Contextual obstacles are crucial to SBM's success, particularly in difficult circumstances like conflict and with program sponsors like the World Bank.

The SBM studies from this time also emphasize how important stakeholders are involved in educational activities. Both benefits and drawbacks come with this engagement, which requires that nations study and develop reasonable policies.

An essential participating component includes teachers [[6], [7], [8]] and local stakeholders [9] SBM is related to the role of the school principal. School-based management will not solve the problems of education, but if a school is under the leadership of an effective, competent principal, the needs of the students, staff, and community will more likely be met as well as leadership at the policy-making and autonomy level of each school.

The recent focus on various concerns of SBM such as empowering schools to address school illnesses (e.g., abesity, chronic asthma, Type 1 diabetes) or children with special needs (e.g.,swallowing), gender, accessibility.

Infused within the ‘topical themes’ that comprised the research front is the delineation of several research methodologies among the recent keywords. Thus, we noted an increased use of qualitative methods, mixed methods, and action research to explore SBM expecially in developing countries.

Concurrently, this analysis also highlights the growing use of multi-factor statistical tests (e.g., SEM, HLM, factor analysis) toward unpack complex interactions between context factors, leadership practices, mediating factors, and learning outcomes. This review identified a rapidly growing body of recent scholarship that illustrates this approach. This research has enabled scholars to develop increasingly refined models of how SBM impacts student achievement.

Table 5 presents some keywords with the same meaning or simply a shorter display were replaced; for example, “School-based Management” changed to “Decentralization” or “School-Based Management” and “Education Reform”, ‘Management’ changed to ‘Education Policy’. The size of the node represents the frequency of the keyword, while the width of the link between two keywords implies the co-occurrence count of the pair. Furthermore, there are also individual terms that appear separately, less forming connections with other words, such as Schools, Education, Human, and Hong Kong. During the research period and with the data provided by the SCOPUS database, there are several keywords associated with the shifts in School-based Management research. These studies are linked to primary schools, focusing on ensuring equity and equality in education. Particularly, school-based management has been employed to support and enhance the effectiveness of educational activities related to children with special needs or illnesses occurring within the school environment. Hong Kong emerges as a prominent location with numerous studies on School-based Management, being a place where comprehensive implementations of School-based Management reforms have taken place.

**Table 5:** 10 keywords with the most co-occurrences in the publications on SBM.

No.	Key words	Occurrence
1	School-based Management	89
2	Decentralization	28
3	Schools	25
4	School Based Management	21
5	Education	18
6	Education Policy	16
7	Human	16
8	Management	16
9	Education Reform	13
10	Hong Kong	13

## 4 Experimental Design, Materials and Methods

### 4.1 Search Criteria and Identification of Sources

The author's source for the documents included in this review was the Scopus database. Scopus was chosen because it uses the same criteria when evaluating papers for inclusion in its index. Additionally, it provides more papers for assessments of social science and education research than the Web of Science [10]. Finally, it offers more sophisticated choices for the export of bibliographic data when compared to Google Scholar.

Despite these benefits, Scopus has a serious flaw. The majority of its coverage is in English-language documents. This is important to know while studying a subject like school-based management, as research papers can be produced in a variety of languages, including Chinese, French, Spanish, Bahasa Indonesia, etc. In light of this, we immediately see that more evaluations of the literature in other languages will be required to produce a comprehensive picture of the literature on SBM on a global scale.

Journal articles, books, book chapters, and conference proceedings were all included in the Scopus document search. There was no time constraint on when a paper might be published; instead, we let Scopus search for pertinent studies from 1984 through 2023. The evaluation's topic was "Bibliometric analysis of school-based management research" papers for SBM that place a focus on content.

The articles that the above-mentioned keyword search resulted in were further examined in the second step. The English language, an unrestricted topic area, an unlimited number of document categories, and an unlimited publishing term were just a few of the screening criteria that were utilized to choose the best documents. This stage saw the advancement of 337 documents to the third phase, eligibility. The third part involved reading the title and abstract of each document from 1984 through 2023. Each participant was given the assignment of reviewing the collection of chosen papers and making a recommendation for their retention or removal. At the end of this phase, there was a discussion among all participants on the rationale for deleting a certain document from the analysis data. The researchers looked at the title, abstract, and on rare occasions the entire manuscript to determine if it should be accepted or rejected. A total of 319 documents from the final dataset were saved as SBM files for bibliometric analysis in the future.

The previously given search query produced papers, which were subsequently further filtered.

In this study, descriptive data were used to compile a list of the essential authors, affiliations, sources, and documents in SBM. Using analytical techniques including co-authorship analysis, citation analysis, and co-occurrence author keywords, it was then possible to link significant information that related authors, sources, affiliations, and documents in the SBM topic. Consistent with the results of other studies, this one used VOS viewer software to analyze the data.

### 4.2 Data Analysis

Scopus, SBM document metadata for the years 1984 through 2023 was exported into a master Excel file. Excel was used to compile a descriptive statistical study of the SBM landscape, including information on growth patterns, regional distribution, and types of research publications. Data were then loaded into VOS viewer, a tool for bibliometric mapping of science, using the master Excel file as a source [11]. Bibliometric analyses carried out in VOS viewer included author co-citation analysis and author and document citation analysis.

VOS viewer now includes both author and document citation analysis as well as author co-citation analysis. Citation analysis is frequently used to find important authors and works in particularly academic domains [12]. To find out how frequently authors and the 348 publications in the review database were cited in other Scopus documents, citation studies were carried out in VOS viewer. These are referred to as "Scopus citations" as they are only made by publications that are indexed by Scopus.

Author co-citation analysis was used to generate "co-citation counts" that were then used in a network map to depict the theoretical organization of the literature [12,13]. Author co-citation [14] refers to the frequency with which two writers are cited collectively in the "reference lists" of papers in the review database. In co-citation analysis, the source of the citation data is an essential distinction. The results are therefore not constrained by the Scopus source index, unlike citation analysis.

Another advantage of co-citation analysis is the ability to "visualize similarities" between writers. According to Small's hypothesis [14], authors who are often co-cited (i.e., mentioned together by other authors) are likely to share similar intellectual interests. Matrixes of co-citation frequencies are produced by VOS viewers as the input for co-citation mapping and the "visualization of similarities" or VOS [11]. This study used author co-citation analysis to show the connections between writers who were often co-cited in the SBM base [11,12].

After the procedure was complete, we produced a data set of 293 articles that could be examined as a Microsoft Excel file. Each data line will include many columns of information, including the author(s), link(s) of the author(s), document name, source type, document description, keywords, number of citations of the document, year of publication, and references.

## Limitations

The first implication we draw from these conclusions is associated with the growth and ever-increasing impact of the scholarship on school-based management. The field of SBM has over the past 60 years reorganized around a conceptual core concerned first and foremost with ‘transfer the power to school”. This suggests the continued relevance of instructional leadership in both policy formulation and school management.

Secondly, it was noteworthy that findings from the geographical analysis and co-word analysis highlighted studies of SBM in Asia and developing countries as part of the ‘research front’. We assert that the development of a globally relevant knowledge base on SBM requires an empirically validated understanding of how school-based management achieves its effects in different institutional and cultural contexts. Studies that address this issue in different national contexts will benefit from a three-pronged research strategy consisting of qualitative, mixed methods, and large-scale cross-cultural comparative research.

However, recent studies leveraging data from the SCOPUS database predominantly use the English language as the main medium of research. These studies span over approximately 40 years, during which research on SBM transitioned to developing countries. Countries implementing SBM also largely benefit from the investment of the World Bank aimed at reforming management and equity in education.

Studies also concentrate on a few countries that consider SBM a reform strategy, a mandatory requirement for educational innovation, such as Hong Kong, Indonesia, and Israel. The research tends to be empirical, exploring the actual conditions to highlight the successes, challenges, and prospects of implementing SBM.

Research also indicates that the interest in applying SBM is primarily focused on primary and secondary education. The involvement role of the community in the school board is perceived from multiple perspectives, but the roles of individual components have not been independently and explicitly explored.

In the future, SBM will no longer be experimental but rather a necessary trend in educational management innovation. Research should also be comprehensive and delve deeper into the subsequent aspects of SBM, such as the effectiveness of school boards and the relationship between participation and educational outcomes. Evaluative studies on the level of engagement, participants' profiles, and conditions for involvement in the school board are crucial for SBM. Does this participation genuinely yield SBM's intended results? What criteria and conditions should the community meet for meaningful participation? What steps should schools and countries take to empower this force? Research on principals, teachers, and students within SBM should also be implemented, comparing across educational levels. The demands of competence for principals and teachers in a school's autonomous environment also need exploration.

## Ethics Statements

Our work does not involve studies with animals and humans.

## CRediT Author Statement

**Vu Thi Mai Huong** Project administration, Conceptualization, Methodology, Softwar, Writing- Original draft preparation, Writing- Reviewing and Editing, **Hoang Thi Kim Hue** Supervision, Visualization, Writing - Original Draft, Resources, **Dao Thi Minh Chau** Visualization, Writing - Original Draft, Formal analysis, Methodology, **Nguyen Thi Minh Nguyet** Visualization, Writing - Original Draft, Resources, Investigation, **Trịnh Thi Quy** Visualization, Investigation, Formal analysis, **Nguyen Quoc Tri** Supervision, Investigation, Formal analysis.

## Data Availability

SBM 1983-2023 rawdata (Original data) (Mendeley Data). Dataset about SBM from 1983-2023 (Original data) (Mendeley Data).
